# Effects of Aging and Fitness on Hopping Biomechanics

**DOI:** 10.3390/ijerph192013696

**Published:** 2022-10-21

**Authors:** Horacio Sanchez-Trigo, Jochen Zange, Wolfram Sies, Jonas Böcker, Borja Sañudo, Jörn Rittweger

**Affiliations:** 1 Department of Physical Education and Sports, University of Seville, 41013 Sevilla, Spain; 2 German Aerospace Center (DLR), Institute of Aerospace Medicine, 51147 Cologne, Germany; 3 Department of Pediatrics and Adolescent Medicine, University Hospital Cologne, 50931 Cologne, Germany

**Keywords:** physical fitness, sedentary behavior, aging, biomechanics, stiffness, muscle power

## Abstract

Physical exercise promotes healthy aging and is associated with greater functionality and quality of life. Muscle strength and power are established factors in the ability to perform daily tasks and live independently. Stiffness, for mechanical reasons, is another important constituent of running performance and locomotion. This study aims to analyze the impact of age and training status on one-legged hopping biomechanics and to evaluate whether age-related power decline can be reduced with regular physical exercise. Forty-three male subjects were recruited according to their suitability for one of four groups (young athletes, senior athletes, young controls and senior controls) according to their age (young between 21 and 35, vs. older between 59 and 75) and training status (competing athletes vs. non-physically active). The impact of age and training status on one-legged hopping biomechanics were evaluated using the two-way analysis of variance (ANOVA) method. Significant differences among groups were found for hopping height (*p* < 0.05), ground contact time (*p* < 0.05), peak ground reaction force (*p* < 0.05) and peak power (*p* < 0.01). No differences among groups were found in ground-phase vertical displacement and vertical stiffness (*p* > 0.05). Young athletes and older non-physically active people achieved the best and worst performance, respectively. Interestingly, there were not any differences found between young non-physically active people and senior athletes, suggesting that chronic training can contribute to partly offset effects that are normally associated with aging.

## 1. Introduction

Current demographic data show a significant population aging in developed countries, leading to increased health care costs [1]. Preventing mobility limitations and maintaining independent functioning in the aging population is of major public health importance [2]. One of the factors associated to age-associated decline in mobility is the decline in muscle force and power, which is aggravated by a sedentary lifestyle [3]. Both sedentarism and aging cause a decline in muscle performance and functionality. Thus, it is of interest to assess separately the respective contributions of sedentarism and aging to better understand this process, and to evaluate to which degree can a physically active life compensate age-associated decline in muscle power. Master athletes are particularly interesting to study these processes. These are individuals who train to compete in athletic events at a high level beyond a typical sports retirement age [4]. Master athletes can be considered as rare examples of aging without the common confounder of increased sedentarism at older age [4]. Previous research on master athletes has shown a clear effect of age and athletic specialization on muscle power since, while aging is associated with a 40% reduction in jumping power from the 3rd to the 7th decade of life, sprint-trained master athletes have greater jumping power than endurance master athletes [4,5,6]. To a large extent, the age-related decline in jumping power is explicable by age-effects on body composition [7]. However, to the best of our knowledge, no previous study has yet compared age-related effects on muscle power between master athletes and a cohort matched in age, height and weight that is non-physically active. In this study, we compare the jumping performance and biomechanics of athletes and ordinary active non-athletes control subjects, both at a young age and in elder subjects typically affected by aging processes. Our aim is to quantify how far the plyometric performance of elite master athletes is superior above age matched non-athletes, and how far the jumping biomechanics of athletes with respect to non-athletes is affected by aging and fitness. To answer this research question, the effects of age and training status on jumping biomechanical parameters will be assessed. It is our hypothesis that a prolonged engagement in exercise is related to a reduced age-related decline in muscle power and performance.

## 2. Materials and Methods

### 2.1. Participants

This study was conducted within the MALICoT project, which was designed to compare intramuscular connective tissue between young athletes, young non-physically active people, senior athletes and senior non-physically active people. Specifically, the study targeted power athletes (jumpers and sprinters) for the athlete groups. For the recruitment process, the study was advertised via social media and on the DLR website (www.dlr.de/me/en/desktopdefault.aspx/tabid-15377/ accessed on 20 September 2022). An online questionnaire was filled out by interested subjects. Activity levels were quantified using the Freiburger questionnaire for physical activity [8,9] and the subjects’ energy expenditure was estimated, in terms of metabolic equivalents of task (METs). The questionnaire included questions to check criteria for inclusion and exclusion. Inclusion criteria were (a) age either between 20 and 35 for the young groups and age between 60 and 75 for the senior groups; (b) ≥4 h per week training and regular competition in sprint running or jumping events for the athletic groups, and ≤25 METs per week spent in exercise for the non-physically active group; (c) male sex; and (d) ability to provide informed consent (all groups). People were excluded when diagnosed with diabetes, when they had contraindications against magnetic resonance imaging or against muscle biopsy, or when they had experienced injuries or musculoskeletal disorders likely to interfere with the testing protocol. 

All participants provided written informed consent prior to participating in this study. The experimental protocol was approved by the Ethical Committee of the Ärztekammer Nordrhein in Düsseldorf, Germany (ref. no. 2018269). The study was prospectively registered on the German register of clinical trials (www.drks.de accessed on 20 September 2022) with registration number DRKS00015764.

### 2.2. Sample Size

As mentioned above, this study is part of the MALICoT project, whose main goal is to investigate endomysium and perimysium content as a function of age and training state in humans. The lack of information on age- and training-state dependency of muscle tissue’s elastic modulus makes a statistically-motivated sample size definition difficult. However, a sample size of 12 subjects per each of the four sub-groups (young athletes, young non-physically active people, senior athletes and senior non-physically active people), and, thus, a total of 48 seemed a feasible goal, based on previous experience. On the other hand, preliminary data suggest a variation coefficient of 1.76% for reproducibility. Thus, with such a good reproducibility, this study aimed to allow the estimation of group means and their standard deviation, and to discover effects of age and training state. As stated above, there has been no previous quantitative human study on endomysium thickness. For sample size calculation, we therefore rely on previous research reporting a group difference between 6- and 18-week-old chickens of 1.03 µm with standard deviation of 1.24 µm in endomysium thickness [10]. Using a *t*-test to test the primary hypothesis, and setting α = 0.05 and *β* = 0.2, we arrive at an estimated sample size of 24 subjects per group. The study aimed, therefore, at including 12 subjects per group, and, thus, a total of 48 participants. 

### 2.3. Testing Procedures

Jumping mechanography was employed to assess motor performance. This technique relies on plyometric tests performed on a force platform to evaluate dynamic muscle function and has been found to be a reliable and sensitive measure of mobility performance in elite athletes as well as in frail patients employing both two-legged and single-legged jumps [3,11]. In the present study, a multiple one-leg hopping (M1LH) test using the dominant leg was carried out [12,13,14]. Subjects were instructed to start with shallow hops, increase height to maximum followed by 4 to 5 maximum hops and finally reduce hop height again.

The M1LH test was performed on a force plate (Leonardo Mechanography GRFP, Novotec Medical Inc., Pforzheim, Germany) continuously measuring the vertical ground reaction forces (GRF) at a sampling rate of 800 Hz.

### 2.4. Data Processing

The GRF signals recorded during the M1LH test were analyzed using the module ‘signal’ within the software package R (R Core Team, 2020, Vienna, Austria). Each individual hop was identified by the detecting the flying phases (absence of GRF) and the phases of ground contact (positive GRF). The following variables were calculated for each hop:

- Flight time (*FT*): duration of the flying phase of the hop, that is, time interval in which the subject has no contact with the ground;
- Ground contact time (GCT): interval of time in which the subject’s leg is contact with the ground after the FT;
- Maximum GRF: peak GRF registered during the GCT after landing, and prior to the next hop;
- Hopping height (*HH*). Calculated from flight time using the equation of uniformly accelerated motions [15]:
  $$HH=g\cdot FT^{2}/8$$
  where:
  *g*—gravity acceleration constant (9.81 m/s^2^)
  *FT*—flight time
- Vertical acceleration (*A_V_*) of the center of mass (COM) over time. Calculated from GRF and subject’s body mass [16]:
  $$A_{V}\left(t\right)=\frac{F\left(t\right)-m\cdot g}{m}$$
  where:
  *F(t)*—GRF over time
  *m*—body mass
  *g*—gravity acceleration constant (9.81 m/s^2^)
- Vertical velocity (*V_V_*) of the COM over time. Calculated from the integration in the time domain of the acceleration-time data [16]:
  $$V_{V}\left(t\right)=\int A_{V}\left(t\right)dt+c=\int \frac{F\left(t\right)-m\cdot g}{m}dt+c$$
  where:
  *A_V_(t)*—Vertical acceleration over time
  *F(t)*—GRF over time
  *m*—body mass
  *g*—gravity acceleration constant (9.81 m/s^2^)
  *c*—integration constant

The integration constant (*c*) was based upon the assumption that vertical velocity of the COM was zero at the middle of the GCT; in other words, assuming that COM peak downward displacement is reached at the middle of the GCT between hops [16,17]. Since GRF data is discrete, the previous integration was implemented by summation of the GRF samples.

- Vertical displacement (*D_V_*) of the COM during ground contact. Calculated from numerical double integration in the time domain of the acceleration-time data, or equivalently, from the numerical integration in the time domain of the vertical velocity-time data [16,18]:
  $$D_{V}\left(t\right)=\int V_{V}\left(t\right)dt+c=\iint \frac{F\left(t\right)-m\cdot g}{m}dt\;dt+c$$
  where:
  *V_V_*(*t*)—Vertical velocity over time
  *F*(*t*)—GRF over time
  *m*—body mass
  *g*—gravity acceleration constant (9.81 m/s^2^)
  *c*—integration constant

Since our goal was to determine COM displacement, the integration constant (k) was set to zero at the initial instant [19]. Given that velocity data are discrete, the previous integration was implemented by summation of the velocity samples.

- Max *D_V_*(*t*): Maximum vertical downward displacement of the COM during ground contact (also known as countermovement depth);
- Power output, normalized to subject’s body weight [20]:
  $$P\left(t\right)=\frac{F\left(t\right)\cdot V_{V}\left(t\right)}{m}$$
  where:
  *V_V_*(*t*)—Vertical velocity over time
  *F*(*t*)—GRF over time
  *m*—body mass
- Vertical stiffness (*K*), calculated for each hop as the ratio between the peak GRF and maximum COM displacement, according to the spring–mass model [18,21,22]. Since body size influences stiffness [23], *K* was normalized by body mass for each subject and expressed as kN/m/kg [19,24]:
  $$K=\frac{\max F\left(t\right)}{\max D_{V}\left(t\right)}\cdot m^{-1}$$
  where:
  max *F*(*t*)—Maximum GRF;
  *m*—body mass.

According to the spring–mass model, max *F*(*t*) and max *D_V_*(*t*) coincide in the middle of the ground-contact phase during hopping [18]. In fact, the vertical stiffness parameter, *K*, is only valid if the lower extremity behaves like a simple spring–mass system [19,25]. To evaluate that assumption, the linear correlation between *D_V_*(*t*) and *F*(*t*) was also calculated. Only those hops for which this correlation is r > 0.80 comply with the assumption of spring-like behavior [19,25]. Hops that were unable to meet this criterion were not used for data analysis.

Finally, for each subject the three highest hops (the three hops with the highest HH) were selected, and the averages for all the previously described parameters were computed using these three hops.

### 2.5. Statistical Analysis

The impact of age and training status on the M1LH test was evaluated by comparing all previously described biomechanical parameters using the two-way analysis of variance (ANOVA) with two factors (age × training status). To assess assumptions of homoscedasticity, Levene’s test was performed. Normality was evaluated using Shapiro–Wilk’s test. Group means were compared performing Tukey’s post-hoc test, if a significant main effect was observed. Statistical significance was set at *p* < 0.05. Pearson correlation (r) was employed to measure linear correlation and evaluate the assumption of spring–mass-like behavior. These statistical analyses were performed using Jamovi (The Jamovi project, 2019, Version 1.0).

## 3. Results

### 3.1. Participants Characteristics

Forty-three male subjects completed the study. Twenty-two young subjects (21–35 years old) and twenty-one senior subjects (59–75 years old) were recruited. Among them, ten young subjects and ten senior subjects regularly trained and competed as athletes in sprint or jumping events, while the remaining subjects (twelve young and eleven senior) were only ordinary physically active without performing intensive and specific training like the subjects in the two athletes’ groups do. Thus, four groups were established: young athletes (YA), young controls (YC), senior athletes (SA) and senior controls (SC). Their characteristics are summarized in Table 1.

### 3.2. Biomechanical Parameters

Table 2 shows the descriptive statistics (average ± standard deviation) of the biomechanical parameters calculated for the M1LH test. As described in Section 2.4, these parameters included *HH*, GCT, maximum GRF, maximum *D_V_*, *K* and maximum power.

### 3.3. Hopping Height

A two-way ANOVA was performed to analyze the effect of age and training status on hopping height revealing that there was not a statistically significant interaction between the effects of age and training status (F-value = 0.264, *p* = 0.610). Simple main effects analysis showed that age did have a statistically significant effect on hopping height (F-value = 34.995, *p* < 0.001), and that training status also had a statistically significant effect on hopping height (F-value = 21.823, *p* < 0.001). Tukey’s post-hoc test results for multiple comparisons are shown in Table 3.

### 3.4. Ground Contact Time

A two-way ANOVA was performed to analyze the effect of age and training status on GCT revealing that there was not a statistically significant interaction between the effects of age and training status (F-value = 6.21 × 10 ^−7^, *p* = 0.999). Simple main effects analysis showed that age did not have a statistically significant effect on GCT (F-value = 3.16, *p* = 0.084), although simple main effects analysis showed that training status did have a statistically significant effect on GCT (F-value = 8.30, *p* = 0.007). Tukey’s post-hoc test results for multiple comparisons are shown in Table 4.

### 3.5. Maximum Ground Reaction Forces

A two-way ANOVA was performed to analyze the effect of age and training status on max GRF revealing that there was not a statistically significant interaction between the effects of age and training status (F-value = 3.35, *p* = 0.075). Simple main effects analysis showed that age did have a statistically significant effect on max GRF (F-value = 4.97, *p* = 0.032), and that training status also had a statistically significant effect on max GRF (F-value = 4.56, *p* = 0.040). Tukey’s post-hoc test results for multiple comparisons are shown in Table 5.

### 3.6. Maximum D_V_

A two-way ANOVA was performed to analyze the effect of age and training status on maximum *D_V_* revealing that there was not a statistically significant interaction between the effects of age and training status (F-value = 0.9687, *p* = 0.332). Simple main effects analysis showed that age did not have a statistically significant effect on maximum *D_V_* (F-value = 0.0956, *p* = 0.759), and that training status did not have a statistically significant effect on maximum *D_V_* (F-value = 0.0609, *p* = 0.806). Tukey’s post-hoc test results for multiple comparisons are shown in Table 6.

### 3.7. Vertical Stiffness

A two-way ANOVA was performed to analyze the effect of age and training status on K revealing that there was not a statistically significant interaction between the effects of age and training status (F-value = 3.658, *p* = 0.064). Simple main effects analysis showed that age did not have a statistically significant effect on K (F-value = 0.385, *p* = 0.539), and that training status did not have a statistically significant effect on K (F-value = 1.852, *p* = 0.182). Tukey’s post-hoc test results for multiple comparisons are shown in Table 7.

### 3.8. Maximum Power

A two-way ANOVA was performed to analyze the effect of age and training status on maximum power revealing that there was not a statistically significant interaction between the effects of age and training status (F-value = 0.848, *p* = 0.363). Simple main effects analysis showed that age did have a statistically significant effect on maximum power (F-value = 31.105, *p* < 0 .001), and that training status also had a statistically significant effect on maximum power (F-value = 14.452, *p* < 0 .001). Tukey’s post-hoc test results for multiple comparisons are shown in Table 8.

## 4. Discussion

The goal of this study was to assess the effects of aging and fitness on jumping performance and biomechanical parameters. To do so, four groups (i.e., YA, SA, YC and SC) were established according to their age (young, between 21 and 35, vs. older, between 59 and 75) and fitness status (competing athletes vs. non-physically active).

YA and SC showed the highest (16.6 ± 3.3 cm) and lowest (6.9 ± 2.3 cm) HH, respectively, which differed significantly from the other two groups (YC: 11.8 ± 2.5 cm, SA: 10.7 ± 3.4 cm; all *p* < 0.05). GCT was significantly shorter for YA (275 ± 48 ms) compared to SC (348 ± 48 ms; *p* = 0.014), with no statistical differences between the other groups (YC: 320 ± 50 ms, SA: 303 ± 53 ms; all *p* > 0.05). Maximum GRF was significantly higher for YA (2.87 ± 0.52 kN) compared with the rest of the groups (YC: 2.32 ± 0.57 kN, SA: 2.31 ± 0.31 kN, SC: 2.26 ± 0.30 kN; all *p* < 0.05). Peak power was significantly higher for YA (32.9 ± 6.5 W/kg) compared with the rest of the groups (YC: 25.5 ± 4.8 W/kg, SA: 22.7 ± 4.9 W/kg, SC: 18.1 ± 3.2 W/kg; all *p* < 0.01), and for YC compared to SC (*p* < 0.01). No statistically significant differences among groups were found in maximum *D_V_*, expressed as a percentage of subject’s height (YA: 9.32 ± 1.8%, YC: 9.77 ± 1.7%, SA: 9.73 ± 2.3%, SC: 8.99 ± 1.8%; all *p* > 0.05). No statistically significant differences among groups were found in vertical stiffness, normalized to body mass (YA: 230 ± 86 N/m/kg, YC: 165 ± 49 N/m/kg, SA: 180 ± 59 N/m/kg, SC: 191 ± 55 N/m/kg; all *p* > 0.05).

As expected, the best performance was observed in YA, and the worst performance was registered in SC in the described M1LH test. Interestingly, there were not any differences found between YC and SA, so these results suggest that chronic training could be associated to a counterbalance of effects that are normally associated with aging. Within young participants, YA showed significantly higher GRF and power than YC, while there were no differences in ground contact time, vertical displacement (during countermovement) and stiffness, so it could be hypothesized that higher fitness improves performance by increasing force application and muscle power, but it doesn’t affect the other biomechanical parameters. Within older participants, SA showed a significantly higher performance than SC, although there were no statistically significant differences between the analyzed biomechanical parameters, probably due to the reduced number of participants. Within trained individuals of different age, YA showed significantly higher GRF and power than SA, while there were no differences in ground contact time, vertical displacement (during countermovement) and stiffness, suggesting, therefore, that aging negatively affects force application and muscle power, but it doesn’t affect the remaining biomechanical parameters. Age-related changes in muscle power have been previously reported in the literature [26]. Within sedentary individuals, YC had a better performance than SC probably attributable to a significantly higher muscle power, suggesting again that aging negatively affects muscle power [27]. In conclusion, both aging and sedentarism result in a decreased muscle power in the M1LH test, but lifelong training could be associated to a counterbalance of the effects of aging [28,29,30,31,32].

There are several limitations to the study. First, the number of participants is reduced, which could be limiting the significance of our findings. Second, there were male participants only. Including females might have unveiled other results, as there are major differences between female and male skeletal muscles, including differences in energy metabolism, fiber type composition, and contractile speed [33]. Finally, only sports with a high implication of muscle power (sprinting and jumping) were considered in the participants’ selection. It would be of interest to include other athletic modalities and sports.

Future research directions might include studying differences in muscle architecture and the connective tissue of the muscles to better understand the underlying causes of age-related decline in power and how to optimize physical training to counteract such processes. In the elder athletes, the superior performance may result from both, an intensive training and a genetically determined slower aging process. The number of athletes performing sprint or jumping disciplines in high age is extremely small, and much smaller compared with the more frequent elder endurance runners. The small number of cases could suggest that the conservation of plyometric performance in senior sprinters and jumpers might not only result from adaptation on training but may a have genetical component affecting aging as well. Future studies should analyze genetical characteristics of master athletes to clarify this question. More importantly, further research and action are required to propagate master athletics as a role model and therefore contribute to improve life quality in our aging society.

## 5. Conclusions

Lifelong athletic training can contribute to partly offsetting age-related muscle power decline.

## Figures and Tables

**Table 1:** Participants characteristics.

	Young Athletes	Young Controls	Senior Athletes	Senior Controls
N	10	12	10	11
Height [cm]	178.9 ± 7.7	180.8 ± 6.7	177.6 ± 7.6	176.9 ± 5.8
Weight [kg]	76.2 ± 13.7	75.4 ± 13.0	74.8 ± 8.4	79.8 ± 8.8
Age [years]	23.9 ± 2.3	28.9 ± 4.5	65.1 ± 4.1	66.1 ± 4.8
Activity Level [METs/week]	55.4 ± 22.8	20.4 ± 42.9	94.3 ± 39.5	23.9 ± 13.2

N: number of subjects. METs: metabolic equivalents of task.

**Table 2:** Biomechanical parameters of the multiple one-legged hopping test.

	Young Athletes	Young Controls	Senior Athletes	Senior Controls
Hopping Height [cm]	16.6 ± 3.3	11.8 ± 2.5	10.7 ± 3.4	6.9 ± 2.3
Ground Contact Time [ms]	275 ± 48	320 ± 50	303 ± 53	348 ± 48
Max GRF [kN]	2.87 ± 0.52	2.32 ± 0.57	2.31 ± 0.31	2.26 ± 0.30
Max *D_V_* [%]	9.3 ± 1.8	9.8 ± 1.7	9.7 ± 2.3	9.0 ± 1.8
Vertical Stiffness [N/m/kg]	230 ± 86	165 ± 49	180 ± 59	191 ± 55
Max Power [W/kg]	32.9 ± 6.5	25.5 ± 4.8	22.7 ± 4.9	18.1 ± 3.2

Data reported as average ± standard deviation. GRF: ground reaction forces. *D_V_*: Vertical displacement of the center of mass during ground contact.

**Table 3:** ANOVA results for hopping height.

Comparison	Mean Difference	SE	df	*t*	*p*-Value	Cohen’s d	95% C.I.	
							Lower	Upper
Young − Seniors	5.40	0.913	36.0	5.92	<0.001	1.88	1.09	2.66
Athletes − Controls	4.26	0.913	36.0	4.67	<0.001	1.48	0.747	2.21
Young athletes − Young controls	4.73	1.29	36.0	3.658	0.004	1.644	0.651	2.637
Young athletes − Senior athletes	5.87	1.32	36.0	4.436	<0.001	2.038	0.987	3.090
Young athletes − Senior controls	9.66	1.32	36.0	7.305	<0.001	3.356	2.127	4.586
Young controls − Senior athletes	1.14	1.26	36.0	0.903	0.803	−0.394	−1.286	0.497
Young controls − Senior controls	4.93	1.26	36.0	3.919	0.002	1.712	0.736	2.689
Senior athletes − Senior controls	3.79	1.29	36.0	2.947	0.027	1.318	0.358	2.278

**Table 4:** ANOVA results for ground contact time.

Comparison	Mean Difference	SE	df	*t*	*p*-Value	Cohen’s d	95% C.I.	
							Lower	Upper
Young − Seniors	−28.0	15.8	36.0	−1.78	0.084	−0.564	−1.22	0.0932
Athletes − Controls	−45.4	15.8	36.0	−2.88	0.007	−0.913	−1.59	−0.234
Young athletes − Young controls	−45.4	22.3	36.0	−2.032	0.195	−0.913	−1.85	0.0240
Young athletes − Senior athletes	−28.0	22.8	36.0	−1.227	0.614	−0.564	−1.51	0.3776
Young athletes − Senior controls	−73.4	22.8	36.0	−3.214	0.014	−1.477	−2.47	−0.4804
Young controls − Senior athletes	17.4	21.7	36.0	0.800	0.854	−0.349	−1.24	0.5407
Young controls − Senior controls	−28.0	21.7	36.0	−1.290	0.575	−0.563	−1.46	0.3329
Senior athletes − Senior controls	−45.4	22.2	36.0	−2.041	0.192	−0.913	−1.85	0.0200

**Table 5:** ANOVA results for maximum ground reaction forces.

Comparison	Mean Difference	SE	df	*t*	*p*-Value	Cohen’s d	95% C.I.	
							Lower	Upper
Young − Seniors	0.312	0.140	36.0	2.23	0.032	0.707	0.0421	1.37
Athletes − Controls	0.299	0.140	36.0	2.14	0.040	0.677	0.0141	1.34
Young athletes − Young controls	0.5546	0.198	36.0	2.7979	0.039	1.2576	0.298	2.217
Young athletes − Senior athletes	0.5677	0.203	36.0	2.8018	0.039	1.2873	0.306	2.269
Young athletes − Senior controls	0.6103	0.203	36.0	3.0121	0.023	1.3840	0.395	2.373
Young controls − Senior athletes	0.0131	0.193	36.0	0.0681	1.000	−0.0298	−0.916	0.856
Young controls − Senior controls	0.0557	0.193	36.0	0.2893	0.991	0.1264	−0.760	1.013
Senior athletes − Senior controls	0.0426	0.197	36.0	0.2161	0.996	0.0966	−0.811	1.004

**Table 6:** ANOVA results for maximum *D_V_*.

Comparison	Mean Difference	SE	df	*t*	*p*-Value	Cohen’s d	95% C.I.	
							Lower	Upper
Young − Seniors	0.00186	0.00601	36.0	0.309	0.759	0.0980	−0.545	0.741
Athletes − Controls	0.00148	0.00601	36.0	0.247	0.806	0.0783	−0.565	0.721
Young athletes − Young controls	−0.00443	0.00852	36.0	−0.5201	0.954	−0.2338	−1.147	0.680
Young athletes − Senior athletes	−0.00406	0.00871	36.0	−0.4658	0.966	−0.2140	−1.147	0.719
Young athletes − Senior controls	0.00334	0.00871	36.0	0.3836	0.980	0.1763	−0.757	1.109
Young controls − Senior athletes	0.000375	0.00828	36.0	0.0452	1.000	−0.0198	−0.906	0.866
Young controls − Senior controls	0.00777	0.00828	36.0	0.9384	0.784	0.4100	−0.482	1.302
Senior athletes − Senior controls	0.00740	0.00848	36.0	0.8727	0.819	0.3903	−0.521	1.302

**Table 7:** ANOVA results for vertical stiffness.

Comparison	Mean Difference	SE	df	*t*	*p*-Value	Cohen’s d	95% C.I.	
							Lower	Upper
Young − Seniors	12.3	19.8	36.0	0.621	0.539	0.197	−0.448	0.841
Athletes − Controls	27.0	19.8	36.0	1.36	0.182	0.431	−0.220	1.08
Young athletes − Young controls	64.9	28.1	36.0	2.309	0.115	1.038	0.0931	1.982
Young athletes − Senior athletes	50.2	28.7	36.0	1.748	0.315	0.803	−0.1484	1.754
Young athletes − Senior controls	39.3	28.7	36.0	1.367	0.528	0.628	−0.3157	1.572
Young controls − Senior athletes	−14.7	27.3	36.0	−0.537	0.949	0.235	−0.6532	1.123
Young controls − Senior controls	−25.6	27.3	36.0	−0.937	0.785	−0.410	−1.3011	0.482
Senior athletes − Senior controls	−10.9	28.0	36.0	−0.391	0.979	−0.175	−1.0828	0.733

**Table 8:** ANOVA results for maximum power.

Comparison	Mean Difference	SE	df	*t*	*p*-Value	Cohen’s d	95% C.I.	
							Lower	Upper
Young − Seniors	0.890	0.160	36.0	5.58	<0.001	1.77	0.999	2.54
Athletes − Controls	0.607	0.160	36.0	3.80	<0.001	1.21	0.501	1.91
Young athletes − Young controls	0.754	0.226	36.0	3.33	0.010	1.497	0.5178	2.476
Young athletes − Senior athletes	1.037	0.231	36.0	4.48	<0.001	2.060	1.0061	3.114
Young athletes − Senior controls	1.497	0.231	36.0	6.47	<0.001	2.973	1.8014	4.145
Young controls − Senior athletes	0.283	0.220	36.0	1.29	0.576	−0.563	−1.4592	0.333
Young controls − Senior controls	0.743	0.220	36.0	3.38	0.009	1.476	0.5224	2.430
Senior athletes − Senior controls	0.460	0.225	36.0	2.04	0.192	0.913	−0.0196	1.846

## Data Availability

The data presented in this study are available on request from the corresponding author.
